# Blender interstitial volume: A novel virtual measurement of structural complexity applicable to marine benthic habitats

**DOI:** 10.1016/j.mex.2019.07.014

**Published:** 2019-07-23

**Authors:** Saachi Sadchatheeswaran, Coleen L. Moloney, George M. Branch, Tamara B. Robinson

**Affiliations:** a Department of Biological Sciences and Marine Research Institute, University of Cape Town, Rondebosch, South Africa; b Department of Botany and Zoology, Centre for Invasion Biology, Stellenbosch University, South Africa

**Keywords:** Blender interstitial volume, 3D modeling, Quantitative, Volumetric, Ecosystem engineers

## Abstract

Blender interstitial volume is a novel method that utilizes 3D modeling techniques to accurately and efficiently quantify the volume of interstitial gaps in marine benthic habitats, as well as the space provided by substrate rugosity. This method builds upon the analog methods routinely used on rocky shores and intertidal habitats, including those that measure rugosity, topography, fractals and volume. The method provides a direct Euclidean measurement and uniquely allows retrospective analysis if historical data on species composition are available. Blender interstitial volume allows users to quickly build and measure a large number of samples at no extra cost.

• The program for Blender is free and opensource, and requires no extra equipment.
• Once 3D models of species are made, the entire method takes less than ten minutes to complete.
• Blender interstitial volume is as accurate as Fractal analysis in determining structural complexity on rocky shores, but is more consistent and precise, and better at discerning differences.

## Graphical abstract

**Figure:**
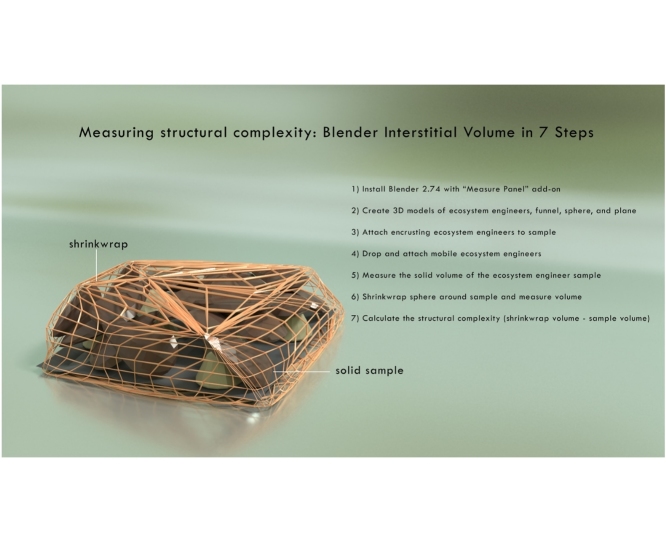

**Specifications Table**

**Table:** 

Subject area:	Environmental Science
More specific subject area:	Marine Ecology – Coastal and Benthic Modeling
Method name:	Blender Interstitial Volume
Name and reference of original method:	The method has been developed using Blender, a 3D modeling software (see Resource availability below). An application of the method appears in Sadchatheeswaran, S., Branch, G.M., Robinson, T.B., 2015. Changes in habitat complexity resulting from sequential invasions of a rocky shore: implications for community structure. Biol. Invasions 17, 1799–1816. The method was further explored and compared favourably against 7 other methods of structural complexity measures in S. Sadchatheeswaran, C.L. Moloney, G.M. Branch, T.B. Robinson, Using empirical and simulation approaches to quantify rival merits of different measures of structural complexity in marine habitats. Mar. Environ. Res. 149 (2019) 157–169. https://doi.org/10.1016/j.marenvres.2019.03.014.
Resource availability:	Original research article: https://doi.org/10.1016/j.marenvres.2019.03.014 Blender 2.74 (Blender Foundation 2012) http://download.blender.org/release/Blender2.74/ Link to 3D models: https://skfb.ly/6AIXY Video tutorial provided (see link for Video 1 below)

## Method details

### Background

Many methods have been applied to measure ‘structural complexity’ in the environment [1,2]. The topic is important in marine benthic habitats because structural complexity and surface ‘roughness’ (rugosity) influence factors such as recruitment and settlement, turbulent exchange of materials such as water, nutrients and oxygen across the surface, space availability for shelter from physical stress and predators, and habitat for feeding [3]. In addition, complexity is known to be correlated with various biological characteristics such as abundance, biomass and diversity [4]. However, none of the methods currently employed to quantify complexity can provide estimates of complexity in a retrospective manner; *i.e*., from reconstructions of historical data on community composition. To address this gap, we developed a novel method, ‘Blender interstitial volume’, that provides a measure of structural complexity [4] in terms of the maximum amount of volumetric space individual organisms can live in (interstitial gaps) or live on (substrate rugosity), which can be applied retrospectively. We used the method in a way that specifically refers to structure created by autogenic ecosystem engineers. These are biota that change properties of the environment in a manner that affects the access to resources by other species, either by the presence of their own bodies or by structures they create, such as tubes [5]. The method is performed using Blender ver. 2.74 [6], an open source software program freely available on blender.org. The method uses Blender's ‘Measure Panel Add-on’ to calculate the volume of samples. Recent versions of Blender do not support the Measure Panel Add-on, but all past versions of Blender currently are available for download. Resources on how to use Blender are available on blender.org, including how to build models, interface with the program and use shortcut keys. All steps described in the procedure below are performed in ‘Wireframe’, ‘Object Mode’ and use the ‘Cycles Render’ engine, unless otherwise specified. An accompanying video tutorial is included for additional clarity (Video 1).

### Procedure (using Windows version)

**Video 1** Full narrated video tutorial of Blender interstitial volume method

### Step 1: Install and setup Blender 2.74 and the Measure Panel Add-on

**Fig. 1:**
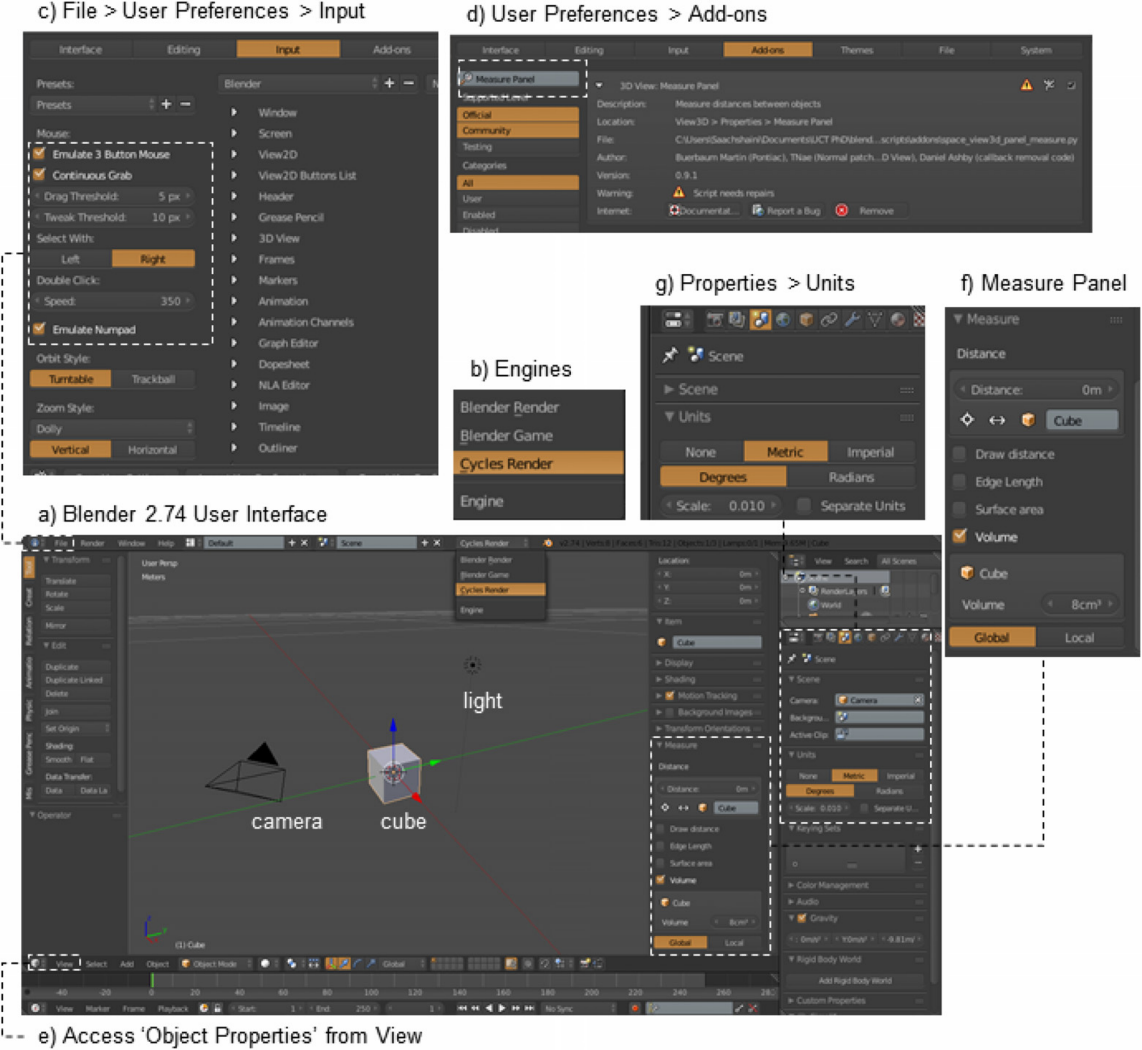
Install and set up Blender 2.74 and the Measure Panel Add-on.
3 Setup user preferences
  a Change engine to ‘Cycles Render’ (Fig. 1b)
  b Click ‘File > User Preferences’
  c In the pop-up window, go to the ‘Input’ tab and check ‘Emulate 3 Button Mouse’ and ‘Continuous Grab’ (Fig. 1c)
  d Also choose 'Select with: Right’. This is the right button on your mouse and this choice allows you to right button-click 3D objects and left button-click everything else in the software, which is the norm in Blender
  e If there is no number pad on your keyboard, check ‘Emulate Numpad’ as well
  f Leave all other default settings
4 Measure Panel Add-on
  a Remain within the User Preferences window and go to the ‘Add-ons’ tab (Fig. 1d)
  b Go to the search box in the top left corner and type in ‘Measure Panel’
  c Check ‘3D View: Measure Panel’
  d Click ‘Save User Settings’ and close the User Preferences window
  e In the main Blender 2.74 window, use the shortcut key ‘N’ to toggle-on the ‘Object Properties’ frame, which appears on the right hand side of the window but has no label. Alternatively, use the 'View' button at the bottom of the window to select 'Properties' and the 'Object Properties' frame will appear (Fig. 1e)
  f Place the cursor on this newly opened frame and scroll down to the very bottom until a button that says ‘Activate’ next to ‘Measure’ is visible (Fig. 1f)
  g Click on the ‘Activate’ button, and open the Measure shelf by clicking the arrow
  h Scroll down if necessary, and uncheck ‘Draw distance’, check ‘Volume’, and make sure ‘Global’ is highlighted instead of ‘Local’
  i As the default cube is the active object, its name and volume are visible as ‘Cube: 8.00000’. This means that the cube has a volume of 8 blender units
5 Change the units to metric
  a Go to the ‘Properties’ frame, the panel on the extreme right hand side of the window, and click on the ‘Scene’ button icon
  b Under the ‘Units’ heading, click on ‘Metric’ and ‘Degrees’, and set the scale to 0.01 (Fig. 1g)
  c Go back to the ‘Object Properties’ frame. Located at the very top of the frame, under ‘Transform > Dimensions’, the dimensions of the default cube (if selected; right button-click to select) should be '2 cm', and under the ‘Measure’ heading at the bottom of the frame, the volume should be '8cm^3^'
    i Note, the scale is very important, or animations that we use later to add ecosystem engineers to the sample might not work

1 Download Blender 2.74 from http://download.blender.org/release/Blender2.74/
  a Download appropriate zip or. exe file for your operating system
  b Transfer file from the downloads folder to a folder of your choice
  c Unzip zip file if necessary
2 Open Blender 2.74
  a Go to the folder, and find ‘blender-2.74-[your operating system]’
  b Double click blender.exe, and open Blender 2.74
  c Click anywhere on the screen to get rid of the welcome and open files screen
  d Blender’s main user interface will be visible, with a default cube, a camera, and a light source (Fig. 1a); the cube will have a yellow outline around it, indicating that it is the current active object, which means that it can be manipulated and measured. Note that while multiple objects at one time can be selected, only one object will be active at any time.

**Fig. 2:**
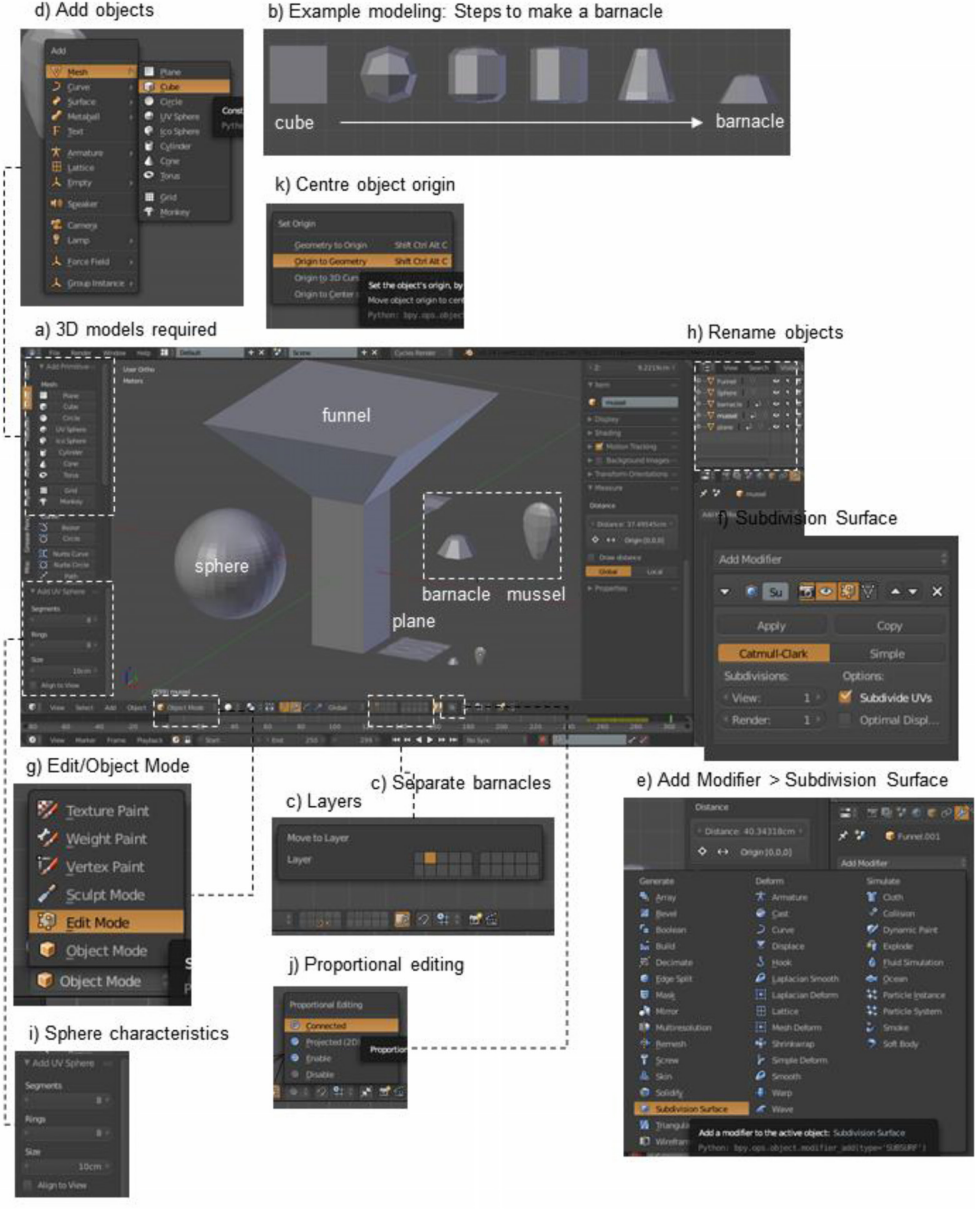
Create or obtain 3D models of ecosystem engineers, plane, funnel, and sphere.

*Step 2: Create or obtain 3D models of ecosystem engineers, plane, funnel, and sphere* (Fig. 2a)

1 The following instructions will go through the simple process of making 3D models of objects. Alternatively, some probable objects (mussels and barnacles) can be downloaded from sketchfab link to models and skip to Step 3a/b – Attach encrusting ecosystem engineers to plane. Specific ecosystem engineers can be commissioned from the first author, Saachi, by emailing her at saachi.sdc@gmail.com
2 In this example, simplified models of an acorn barnacle, *Balanus glandula*, and a mussel, *Mytilus galloprovincialis*, which are both invasive to South Africa, were created
  a Note:
    i It is important to create non-manifold objects, which in Blender means that objects do not contain any faces with more than 4 edges. Manifold objects cannot be measured. Therefore, it’s best to start any model as a cube or sphere
    ii It is also important for objects to have as few vertices as possible, as too many vertices among all the objects can slow or Blender.
    iii As always, please save the Blender file often (File > Save or your system's shortcut key)
3 Build a model of an ecosystem engineer: in this example, a barnacle with an individual volume of 2 cm^3^ (Fig. 2b)
  a Move the default cube to another layer by selecting it and hitting the shortcut key ‘M’ or use the 'Object' button at the bottom of the window to select 'Move to layer' (Fig. 2c). This will cause a window of 20 boxes to appear. Currently the cube is on the first layer, so highlight another layer/box to move it. Near the bottom of the window, the same 20 layers can be seen, with orange dots wherever there are objects. Go to the same layer that the cube is in
  b If a new cube is needed, go to a new layer, use shortcut key ‘Shift + A’, add ‘Mesh > Cube’ (Fig. 2d). You’ll have to change the dimensions from 2 × 2 × 2 m to 2 × 2 × 2 cm in the ‘Object Properties’ frame > Transform > Dimension
  c Barnacles usually have six to eight plates. To achieve this, with the cube selected (yellow outline),
    i Go to the ‘Properties’ frame, the panel on the extreme right-hand side of the window
    ii Choose the 'spanner' icon for 'Modifiers', then 'Add Modifier > Subdivision Surface’ (Fig. 2e) and under 'Subdivisions', set the numbers for both ‘View’ and ‘Render’ to '1' (Fig. 2f). Keep all other default settings
  d Toggle to ‘Edit Mode’ by hitting the ‘Tab’ key (while the cursor is in the 3D view frame), or use the button at the bottom of the window to switch from 'Object mode' to 'Edit mode' (Fig. 2g)
    i Objects are manipulated in edit mode using their vertices, edges, or faces, which can be selected using icons near the bottom left of the ‘3D View window’. Check that the vertices icon is selected
    ii Note: it is easier to edit objects in orthogonal view (shortcut key: Numpad 5) and in front-view (Numpad 1). Other views are side (Numpad 3), or top-view (Numpad 7). The view options can be selected by clicking the 'View' button at the bottom of the window.
    iii Change from solid to wire frame mode with shortcut key ‘Z’, or use the icon for 'Viewpoint shading' (click on the icons to see the options) at the bottom of the window and switch from 'Solid' to 'Wireframe'. This will ensure that all the necessary vertices are selected
  e Add two horizontal cuts into the barnacle with shortcut key ‘Ctrl + R’, then either scroll your mouse-wheel up one notch to get two purple lines OR use shortcut key: ‘PgUp’. Note that you will need to position your mouse correctly on the object so the lines are horizontal and not vertical
    i Left-click and the purple lines will turn orange. Moving your mouse at this stage will move the lines, so left-click again and the lines will stay put
  f With these new lines selected (*i.e*. highlighted in orange), press the shortcut key ‘S' (for Scale) followed by the shortcut key 'Z’ (for the Z-axis - the direction of scaling) and then, without clicking the mouse button, move the mouse away from the object so that the two lines pull apart. When they are almost respectively at the top and bottom of the object, click the mouse button to lock them in place.
  g Make sure none of the vertices are selected by choosing '(De)select All' from the 'Select' button at the bottom of the window. Highlight the top 8 vertices of the object with shortcut key ‘B’, left-click and drag over the vertices (selected vertices will be orange)
  h While in orthogonal and front view (Numpad 5 + 1), position the cursor a short distance away from the object and scale the selected vertices inward by pressing the shortcut key ‘S’ (for Scale) and dragging the mouse toward the object. Click the mouse button. This will shorten the top side of the object. Press 'G' (for Move) and then 'Z' (for the Z-axis) and drag the cursor down. This will reduce the height of the object.
  i In ‘Object Mode’, click the ‘Apply’ button of the ‘Subdivision Surface’ Modifier
  j The resulting barnacle will have an approximate volume of 2 cm^3^. This can be scaled (using shortcut key ‘S’) if necessary.
  k Rename the object as ‘Barnacle’ in the ‘Outliner frame’ (the top right corner of the window) by right-clicking on ‘Cube’ > Rename OR double-click with the left mouse button (Fig. 2h)
4 Model ecosystem engineers: mussel, with an individual volume of 2 cm^3^
  a Move the barnacle to one side (shortcut key ‘G + X’ drag), centre the 3D cursor to the middle of the grid (‘Shift + C’) if necessary, and add a sphere (‘Shift + A’, Add Mesh > UV Sphere)
  b In the toolbar frame (toggle ‘T’), ‘Add UV Sphere’ will appear. Change the number of ‘Segments’ and ‘Rings’ to ‘8’ (Fig. 2i)
  c In ‘Object Mode’, flatten the sphere by going to the side view (Numpad 3) and scaling the sphere along the y-axis (shortcut keys ‘S' then 'Y’) by dragging the mouse toward sphere)
  d In ‘Edit Mode’, click the ‘Proportional Editing’ icon (along the bottom of the window) and choose ‘Connected’ (Fig. 2j). This means all the vertices closest to the one that is manipulated will also be affected
  e Make sure none of the vertices are selected (none are orange) by choosing '(De)select All' from the 'Select' button at the bottom of the window or with shortcut key ‘A’
  f Highlight the sphere’s bottom vertex with shortcut key ‘B’, by left-clicking and dragging over the vertices (selected vertices will be orange). Drag vertically down ('G' then 'Z', drag)
    i Use the scroll wheel on your mouse, or the page up/down buttons to increase the number of vertices that will affected
    ii Left-click when the sphere looks like a mussel
    iii To create a narrowed shape toward the umbo of the mussel, scale the bottom vertex inward with the shortcut key ‘S’
    iv Scale the resulting mussel object’s volume in ‘Object Mode’ to 2 cm^3^ if necessary and rename
5 Create funnel (Fig. 2a) with a base of 10 × 10 cm to deposit mobile ecosystem engineers randomly onto the surface of the plane, and stop any engineers from falling off the plane
  a In ‘Object Mode’, add a cube and change its dimensions to 10 × 10 × 20 cm in the ‘Object Properties’ frame to get a tall rectangular prism
  b In ‘Edit Mode’, disable ‘Proportional Editing’ if necessary, highlight the top four vertices and extend them up (shortcut key ‘E’), left-click and scale them out (S, drag mouse away from prism). The size of this section does not matter, but allow enough space for 20 mussels
  c In ‘Object Mode’, rename this object as ‘Funnel’
6 Add plane (Fig. 2a) to replicate the primary substrate of the quadrat sample
  a In ‘Object Mode’, add a plane (Shift + A, Mesh > Plane) and change its dimensions to 10 × 10 cm
  b If the plane should have bumpy topography, in ‘Edit Mode’, subdivide (shortcut key: ‘W’ > Subdivide) the plane with 10 cuts in the ‘Tools’ frame, and add a fractal of 0.01
7 Add Sphere (Fig. 2a) to shrinkwrap around the entire complete sample and help calculate the empty volume within and immediately surrounding a sample
  a Add a sphere, and in the ‘Tools’ frame, change the change the number of ‘Segments’ and ‘Rings’ to ‘32’, and the size to ‘20cm’
8 Centre origins of all five objects (barnacle, mussel, funnel, plane, and sphere)
  a Highlight (B, drag) all the objects and use the shortcut keys ‘Ctrl + Alt + Shift + C’ > Set Origin > Origin to Geometry (Fig. 2k)

**Fig. 3:**
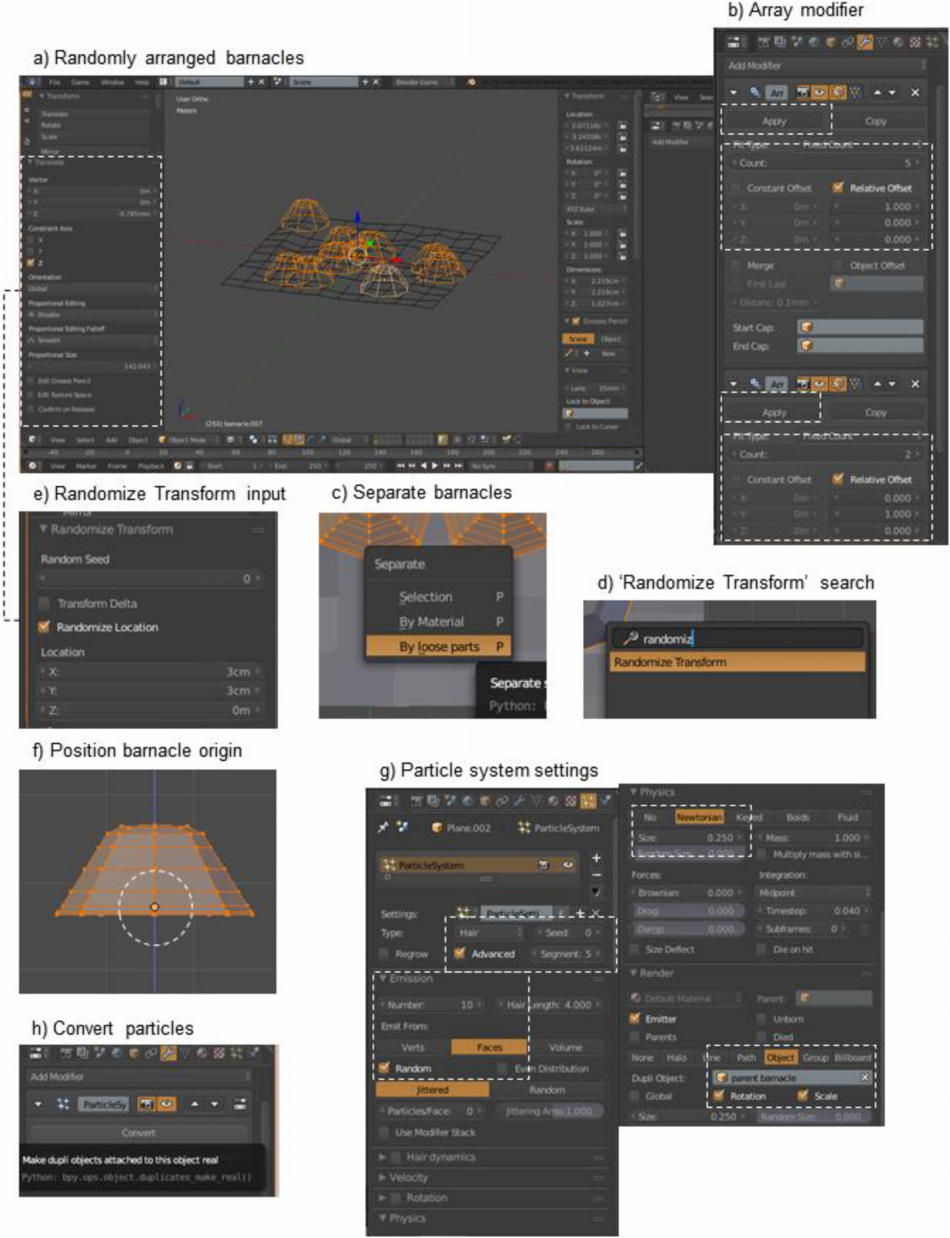
Arrange and attach encrusting ecosystem engineers (*e.g*. barnacles) to plane using a basic or advanced approach.

*Step 3a: Attach encrusting ecosystem engineers (bed of barnacles) to plane – basic approach* (Fig. 3a)

1 Select barnacle (left-click) and plane and place in the same layer
  a Position barnacle on top of the plane (check in top view: Numpad 7)
2 Duplicate barnacle to match historical or current sample data; in this example, ten barnacles of the same size will be included in the sample
  a Duplication can be achieved in multiple ways in Blender. To duplicate one barnacle at a time, use shortcut keys (Shift + D) and drag to place barnacles directly on to plane where appropriate
    i Note: It’s advised to do this in top view (Numpad 7) so that all the barnacles will stay at the same height on the z-axis
  b If there is a large number (n) of objects, they can be duplicated using arrays (Fig. 3b): in the ‘Properties’ frame, select the icon ‘Modifiers’ > Array; Fit type = 10
  c Check ‘Apply’; this array of barnacles is now one object and needs to be separated into ten individual barnacles
  d In ‘Edit Mode’, select the barnacles, and separate them using shortcut keys (P > By loose parts) (Fig. 3c)
  e In ‘Object Mode’, give each barnacle a separate origin (Shift + Ctrl + Alt + C > Origin to Geometry)
3 Arrange all barnacles within plane boundaries in specific layout (*e.g*. if based on sample photographed in the field)
  a Select individual barnacles and, in top-view, moving them with shortcut key (G)
4 Alternatively, randomize the location of each barnacle
  a Select all 10 barnacles and press the space bar
  b Search for ‘Randomize Transform’ (Fig. 3d) and press shortcut key (Enter); controls will appear in the ‘Tools frame’ (T)
  c Randomize barnacle locations by 2–4 cm in the x and y direction (Fig. 3e)
  d Move barnacles back onto plane if necessary
5 Arrange barnacles onto plane surface
  a Select all the barnacles, moving them over the plane if necessary
  b In ‘front view’ (Numpad + 1), move the height of the barnacle bed (G + Z) so that they’re sitting on the plane

*Step 3b: Attach encrusting ecosystem engineers (barnacles) to plane – advanced approach*

Note: If the sample includes numerous encrusting engineers, and/or the primary substrate is very rough or bumpy and this topography is important enough to include in the model, then it is recommended to attach barnacles and the like using a ‘Particle System’. This step can also be used to attach barnacles to secondary substrates, like mussels, and for other species that attach to substrates at specific points and with specific orientations.

1 Prepare the barnacle to generate particles (clones of the barnacle) on the plane
  a In ‘Edit Mode’, position the origin of the barnacle at the very bottom of the barnacle so that the particles will lie flush on the plane (Fig. 3f)
  b Rename this barnacle: ‘parent barnacle’
2 Select plane and set up particle settings
  a In the ‘Properties’ frame, click on the icon ‘Particles’, click ‘New’, and input the following settings (Fig. 3g)
  b Type: Hair; check advanced box; number (of hairs): 10
  c Emission > Emit from: faces with random distribution
  d Physics > Size: 0.25
  e Render > ‘Object’ > ‘Dupli object’: ‘parent barnacle’ and check ‘rotation’ and ‘scale’
  f If the barnacles are in the wrong orientation, select the parent barnacle, and in ‘Edit Mode’, rotate parent barnacle 90 degrees clockwise (Front view: Numpad  + 1; ‘R + 90’) so that all barnacle particles are lying perpendicular and flush to the plane
  g Barnacle particle locations can be randomized using ‘Seed’ at the top of the particle settings
3 Convert the barnacle particles to normal objects
  a With the panel selected, go to the ‘Properties’ frame > ‘Modifiers’ > Convert (Fig. 3h)
4 Measure and adjust volume of the barnacles as required
  a In the ‘Properties’ frame, delete particle system and the parent barnacle
  b Position individual barnacles on the plane so they lie within the boundaries
  c Check one of the barnacles to ensure the volume has stayed at the desired size (2 cm^3^ in this example) and adjust scale of barnacles if necessary

*Step 4: Drop ‘mobile’ ecosystem engineers (mussel) onto plane*

**Fig. 4:**
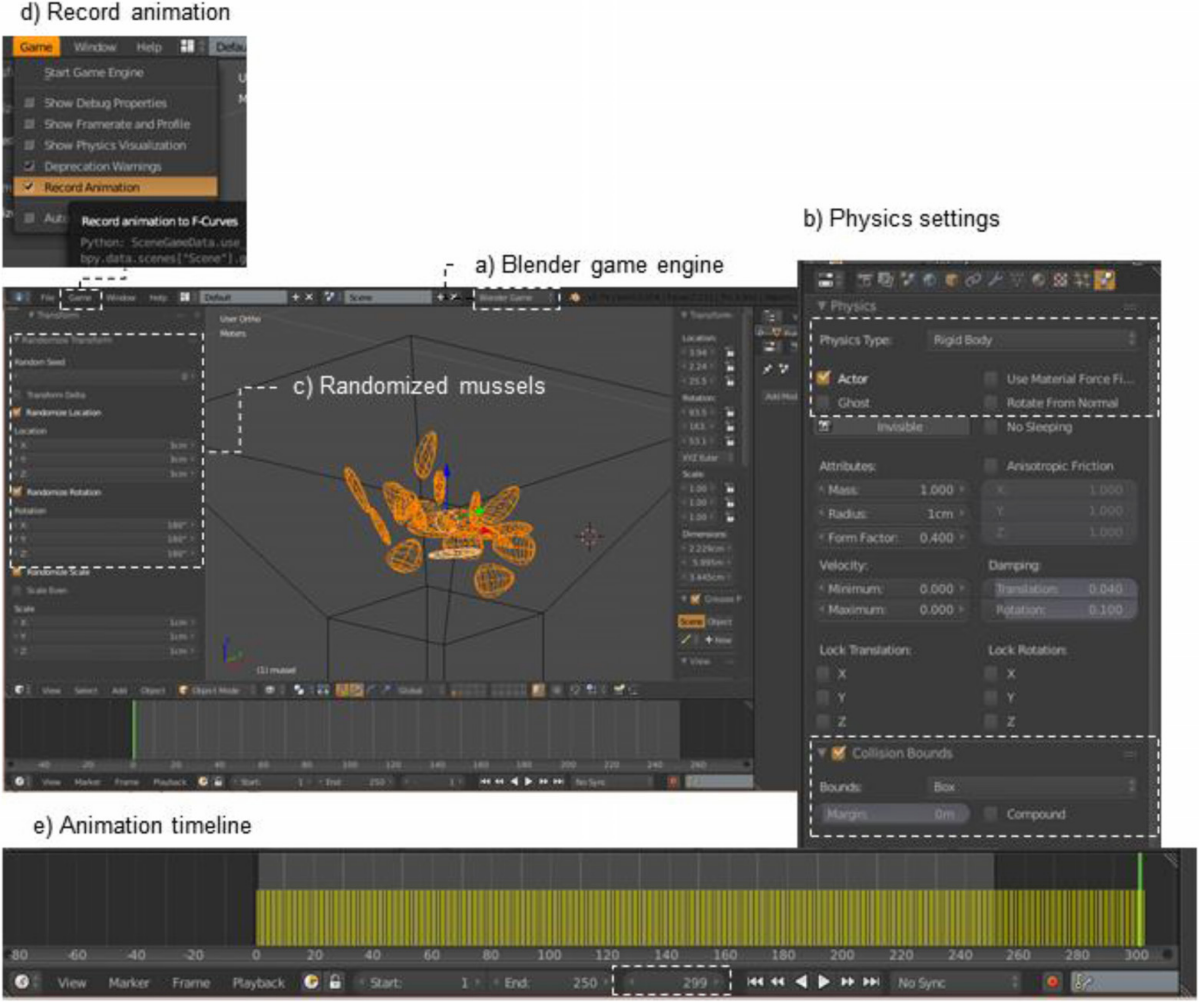
Randomize and drop mobile ecosystem engineers (*e.g*. mussels) onto plane.
  b Physics > Physics Type: Rigid Body
  c Check ‘Collision Bounds’ > Bounds: Convex Hull, Margin: 0 m
  d Test run animation (shortcut key: ‘P’); end animation with ‘Esc’
3 Duplicate the mussel object to match historical or current sample data
  a This example uses 20 mussels, duplicated using the array modifier;
  b Apply array modifier(s) in ‘Object Mode’ and separate mussels in ‘Edit Mode’, (shortcut key: ‘P’ > Separate by Loose Parts)
  c In ‘Object Mode’, give each mussel a separate origin (‘Shift + Ctrl + Alt + C’ > Origin to Geometry)
4 Randomize mussels
  a Select all the mussels
  b Randomize (shortcut keys: ‘Space Bar’ > type ‘Randomize transform’)
  c Set a randomized rotation and location of each mussel by 3 cm and 180° in all directions (Fig. 4c)
5 Run animation
  a In the menu bar, check ‘Record animation’ under ‘Game’ (Fig. 4d)
  b Play the animation (shortcut key: P) until all the mussels are lying on the plane and have stopped moving
  c Escape the animation (shortcut key: Esc) and find the end of the animation in the ‘Timeline frame’ > Current Frame: 300 (Fig. 4e)

1 Position funnel and mussel
  a Move the funnel and mussel to the same layer as the plane and barnacle
  b Place the funnel directly over the panel, so that the plane is inside the funnel
  c Select mussel object and place it in the top part of the funnel
2 Animate mussel object
  a Change engine to ‘Blender Game’ (Fig. 4a), go to ‘Properties’ frame > ‘Physics’, and input the following settings (Fig. 4b)

Step 5: Measure solid volume of sample

**Fig. 5:**
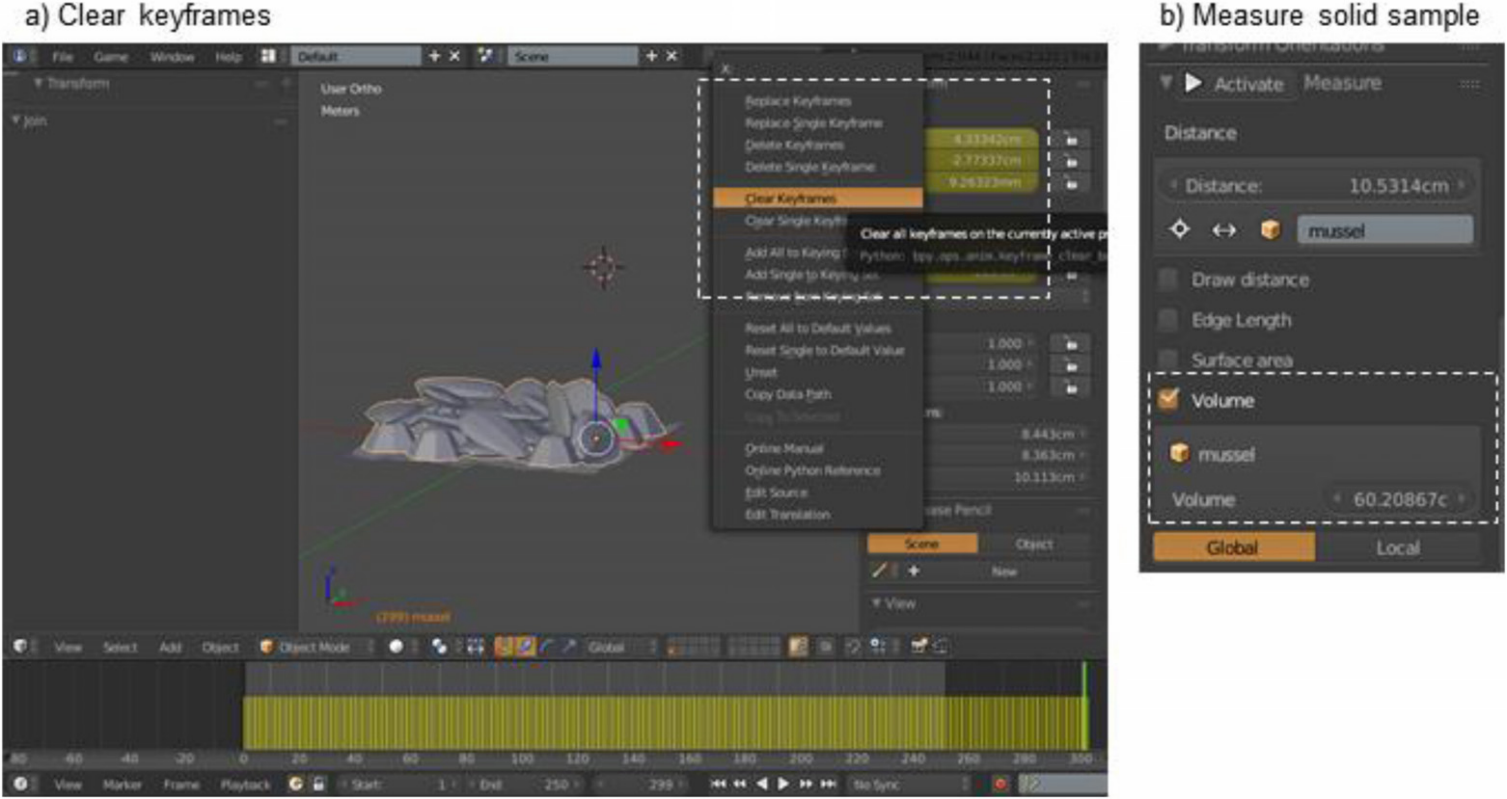
Measure solid volume of sample.
  b Select ‘Clear keyframes’; the bars should be grey
4 Record solid volume of mussels and barnacles together
  a Centre the origin of the sample (‘Shift + Ctrl + Alt + C’ > Origin to Geometry)
  b Measure volume: in this example, it is 60.21 cm^3^ (Fig. 5b)
    i Note: it’s important to measure volumes near the origin of Blender’s grid, as an error in the Measure Panel script allows the volume of objects to change slightly based on their location within 3D space. This error is more pronounced with small-scale objects

1 Move funnel to another layer
  a (shortcut key: ‘M’), choose one of the other 20 boxes
2 Join all ecosystem engineers into one object
  a Select all ecosystem engineers on panel, and with one highlighted yellow (the active object) and all others orange, join them as one object with shortcut keys (Ctrl + J)
    i Note: do not include the panel
3 Clear object’s keyframes
  a Right-click on the yellow bars under both location and rotation in the Object Properties Frame (Fig. 5a)

*Step 6: Measure shrinkwrap volume*

**Fig. 6:**
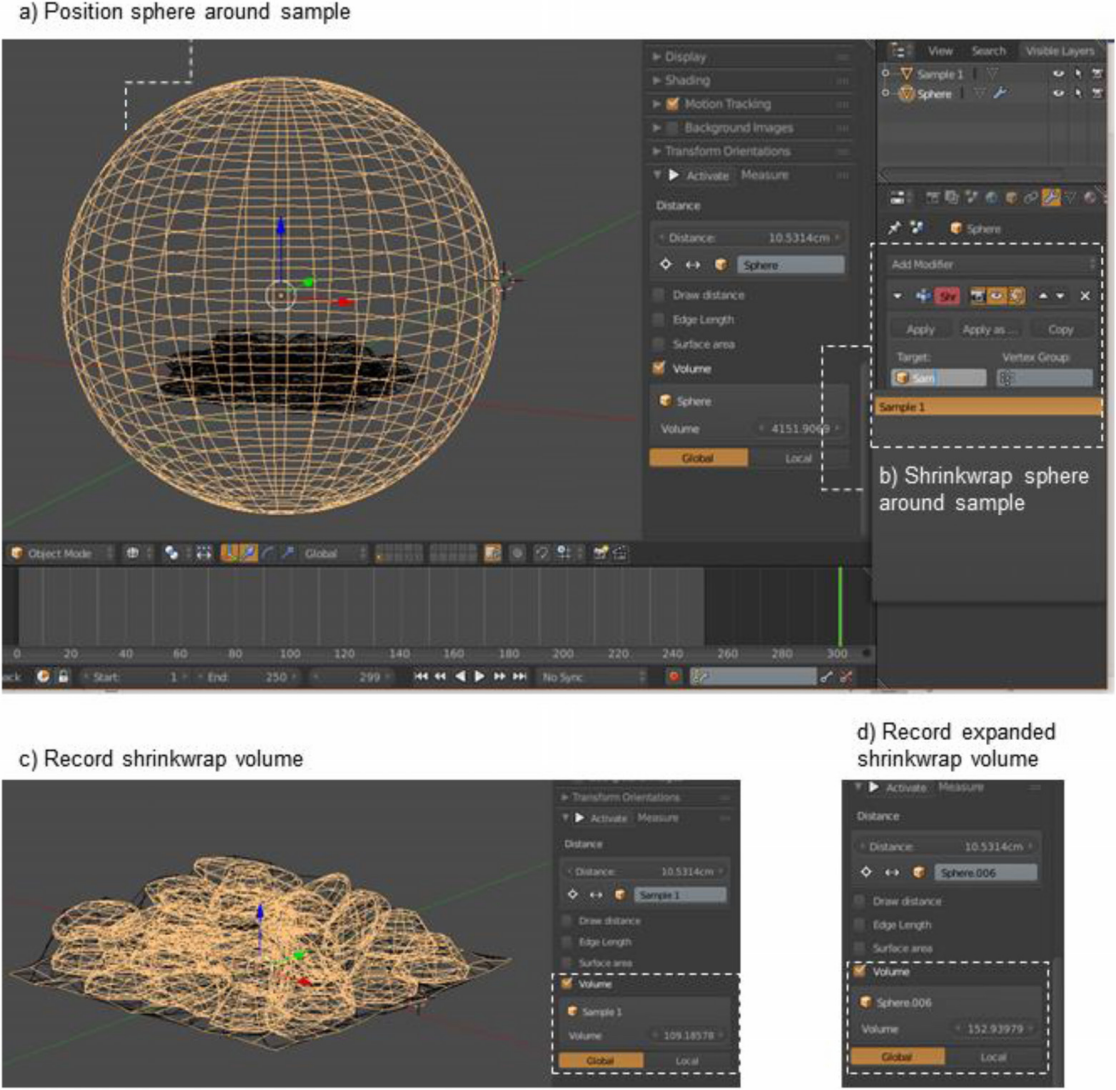
Shrinkwrap sphere around sample, and then measure shrinkwrap and expanded shrinkwrap volumes.
3 Shrinkwrap sphere around Sample 1 (Fig. 6b)
  a In the ‘Properties’ frame, click the ‘Modifier’ icon and add the ‘Shrinkwrap’ modifier
  b Set the ‘Target’ as ‘Sample 1’, and click ‘Apply’
4 Record the volume of the shrinkwrapped sphere in the Measure Panel
  a In this example, this is 109.19 cm^3^ (Fig. 6c)
    i Note: this volume will be dependent on how the ecosystem engineers pack together, and can be highly variable
5 Expand shrinkwrapped sphere for epifauna on the top and sides of the sample
  a Select the shrinkwrapped sphere and go to ‘Edit Mode’
  b Use the following shortcut keys:
    ‘E + Enter + S + 1.1 + X + Enter + E + Enter + S + 1.1 + Y + Enter + E + Enter + S + 1.05 + Z + Enter’
    i Note: this process can be approximated by multiplying the interstitial volume by 1.30, but the final result will not be as accurate
  c Separate the new, expanded shrinkwrap object from original (shortcut keys: ‘P > Selection’)
6 Measure the “expanded shrinkwrap” object in the Measure Panel
  a In ‘Object Mode’, centre the origin of expanded shrinkwrap (‘Shift + Ctrl + Alt + C’ > Origin to Geometry)
  b The expanded shrinkwrap in this example is 152.93 cm^3^ (Fig. 6d)

1 Join ecosystem engineer object and panel
  a Join with ‘Ctrl + J’ and rename final object as “Sample 1”
2 Place sphere around Sample 1
  a Move into the working layer with ‘M’
  b Shift sphere in 3D space with ‘G’ and mouse; sphere should encompass Sample 1 completely (Fig. 6a)

*Step 7: Calculate structural complexity, or Blender interstitial volume, of sample*

1 Blender interstitial volume = ‘expanded shrinkwrap’ volume - solid volume of Sample 1 = 92.72 cm^3^

## Conclusion

Blender interstitial volume has been utilized successfully in two different studies thus far. The first, by the authors of this paper compared the structural impacts of different invasive ecosystem engineers that dominated a rocky intertidal setting near Cape Town, South Africa during the course of three sequential invasions from 1980 to 2012 [4]. The second study compared the impacts of ecosystem engineers on artificial versus natural coastal defenses in Penang, Malaysia [7]. In both studies, Blender interstitial volume demonstrated that an increase in structural complexity was correlated with an increase in species diversity and was able to discern differences in complexity associated with different ecosystem engineers and zonation.

To further validate the use of Blender interstitial volume, it was compared against seven other methods of measuring structural complexity in a companion paper to this methodological paper [8], using seven metrics including (1) correlations among comparable measures; (2) consistency; (3) accuracy; (4) precision; (5) discrimination among configurations of objects; (6) discernment of complexities among zones on rocky shores; and (7) practicality. In addition to volume, the methods also measured rugosity, surface area, and fractal dimensions. While the most commonly used method, Substrate rugosity index, otherwise known as the chain-link method, was the quickest and cheapest method to use, it was also one of the least accurate and had trouble discerning differences in complexity among variable structures [8]. Surface area measures such as fractal analysis demonstrated great accuracy and precision but were difficult to execute. A direct measure of interstitial volume that did not require computational modelling was also tested, but proved prone to human error, and could not be used to analyze complexity from historical datasets. Out of all eight methods, Blender interstitial volume was the most successful and scored the highest or second highest points in nearly all categories [8]. The Blender interstitial volume method was the only method that measures structural complexity quickly and accurately, correlates well with biological data, and can be applied to both current and historical sets of data.
